# Combinations of Vitamin A and Vitamin E Metabolites Confer Resilience against Amyloid-β Aggregation

**DOI:** 10.1021/acschemneuro.2c00523

**Published:** 2023-02-02

**Authors:** Priyanka Joshi, Sean Chia, Xiaoting Yang, Michele Perni, Justus M. Gabriel, Marshall Gilmer, Ryan Limbocker, Johnny Habchi, Michele Vendruscolo

**Affiliations:** † Centre for Misfolding Diseases, Department of Chemistry, University of Cambridge, Cambridge CB2 1EW, U.K.; ‡ The California Institute for Quantitative Biosciences, Department of Nutritional Sciences and Toxicology, University of California, Berkeley, California 94720, United States; § Department of Chemistry and Life Science, United States Military Academy, West Point, New York 10996, United States

**Keywords:** metabolite homeostasis, protein aggregation, chemical chaperones, chemical kinetics

## Abstract

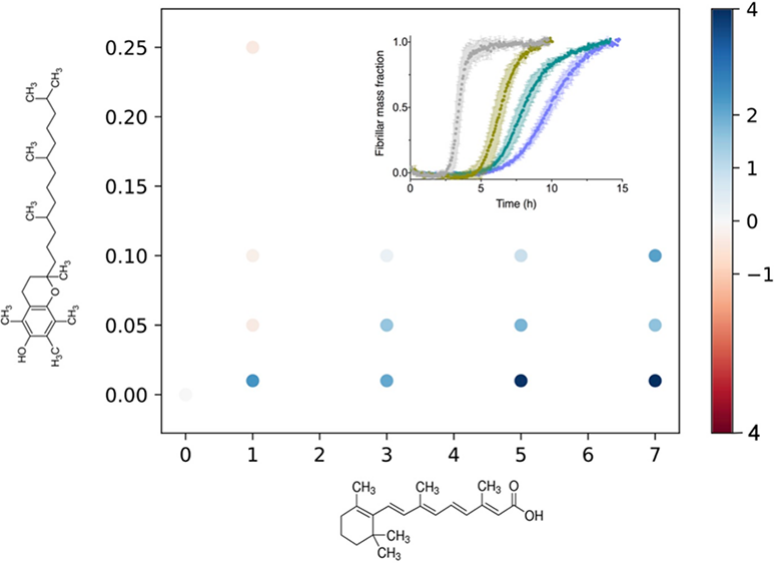

Alzheimer’s disease is characterized by the presence in the brain of amyloid plaques formed by the aberrant deposition of the amyloid-β peptide (Aβ). Since many vitamins are dysregulated in this disease, we explored whether these molecules contribute to the protein homeostasis system by modulating Aβ aggregation. By screening 18 fat-soluble and water-soluble vitamin metabolites, we found that retinoic acid and α-tocopherol, two metabolites of vitamin A and vitamin E, respectively, affect Aβ aggregation both in vitro and in a *Caenorhabditis elegans* model of Aβ toxicity. We then show that the effects of these two vitamin metabolites in specific combinations cancel each other out, consistent with the “resilience in complexity” hypothesis, according to which the complex composition of the cellular environment could have an overall protective role against protein aggregation through the simultaneous presence of aggregation promoters and inhibitors. Taken together, these results indicate that vitamins can be added to the list of components of the protein homeostasis system that regulate protein aggregation.

## Introduction

Alzheimer’s disease (AD) is the leading cause of dementia, a condition that affects over 50 million people worldwide and puts an enormous strain on healthcare systems.^1^ This disease is characterized by the accumulation of extracellular plaques formed of the amyloid-β peptide (Aβ) and of intracellular tangles formed by the tau protein.^2−5^ Because of the toxicity associated with protein aggregation, living systems have evolved complex quality control mechanisms, collectively known as the protein homeostasis system, to control the presence of protein aggregates.^4,6,7^ These mechanisms enable the regulation of protein synthesis, trafficking, interactions, and degradation and involve a wide range of cellular components, including enzymes,^8^ molecular chaperones,^7^ metabolites,^9,10^ and lipids,^11^ which we are only beginning to understand in detail.

More generally, the protein homeostasis system is part of the wider cellular homeostasis system, which regulates all the cellular components, including lipids, carbohydrates, and metabolites. Increasing evidence suggests the presence of a close interplay between the lipid homeostasis and protein homeostasis systems, with different types of lipids having a wide range of effects on protein aggregation.^12−14^ One intriguing aspect of this interplay is the principle of “resilience in complexity”, which states that the opposite effects of different lipids on protein aggregation can cancel each other out, resulting in a zero-sum effect, so that lipid membranes of complex composition effectively buffer the aggregation-promoting roles of certain lipid types.^15^

In this work, we investigate whether a similar principle could be present for vitamins, with the aim of revealing a possible level of interplay between the metabolite homeostasis and the protein homeostasis systems. As a proof of principle, we focused on Aβ, as its abnormal aggregation is one of the underlying causes of AD.^2−5^ We considered 18 fat-soluble and water-soluble vitamin metabolites (Table 1) as vitamin homeostasis has been reported to be dysregulated in aging and in AD,^16^ and vitamin metabolites have been shown to affect Aβ aggregation.^17−20^ Further, we asked what are the microscopic mechanisms by which these vitamin metabolites act to modulate the aggregation of Aβ42. Finally, we show that the combination of an accelerator and an inhibitor cancels out their individual effects on the aggregation of Aβ42.

**Table 1:** List of the Fat-Soluble and Water-Soluble Vitamin Metabolites Tested in the In Vitro ThT-Based Screening Assay.

vitamin metabolite	vitamin group	cellular location (HMDB)		dysregulation in AD (plasma/CSF levels)	effect on Aβ42 aggregation
cobalamin	vitamin B12	membrane (predicted from log P)		decrease	acceleration
cholecalciferol	vitamin D3	cytoplasm, extracellular, membrane, mitochondria		decrease	acceleration
ergocalciferol	vitamin D2	cytoplasm, extracellular, membrane, mitochondria		decrease	acceleration
menaquinone	vitamin K2	not known		decrease	inhibition
menadione	vitamin K3	not known		decrease	inhibition
retinoic acid	vitamin A	cytoplasm, extracellular, membrane, nucleus, endoplasmic reticulum		decrease	inhibition
α-tocopherol	vitamin E	cytoplasm, extracellular, membrane		decrease	acceleration
4-aminobenzoic acid	vitamin B	information not available		not known	no effect
biotin	vitamin B7, vitamin H	cytoplasm, extracellular, mitochondria, nucleus		decrease	no effect
folic acid	volate, vitamin M	extracellular		decrease	no effect
niacinamide	vitamin B3	extracellular		decrease	no effect
d-pantothenic acid hemicalcium salt	vitamin B5	extracellular, mitochondria		decrease	no effect
pyridoxal hydrochloride	vitamin B6 group	extracellular		decrease	no effect
pyridoxamine dihydrochloride	vitamin B6 group (biologically active form)	extracellular		decrease	no effect
pyridoxine hydrochloride	vitamin B6 group	extracellular		decrease	no effect
(−)-riboflavin	vitamin B2	extracellular		not clear	no effect
thiamine hydrochloride	vitamin B1	extracellular, membrane (predicted from log P), mitochondria		decrease	no effect
(+/−)-α-lipoic acid	vitamin-like antioxidant	cytoplasm, extracellular, membrane (predicted from log P)		not clear	no effect

Our results suggest that vitamin metabolites may assist proteins to remain in their soluble state and that a homeostatic balance of vitamin metabolites and proteins underlies a robust molecular environment in a cell. Taken together, our study provides evidence that metabolite homeostasis may act to stabilize proteins against aggregation.

## Results

### An In Vitro Screen of 18 Vitamin Metabolites Identifies Modulators of Aβ42 Aggregation

**Figure 1:**
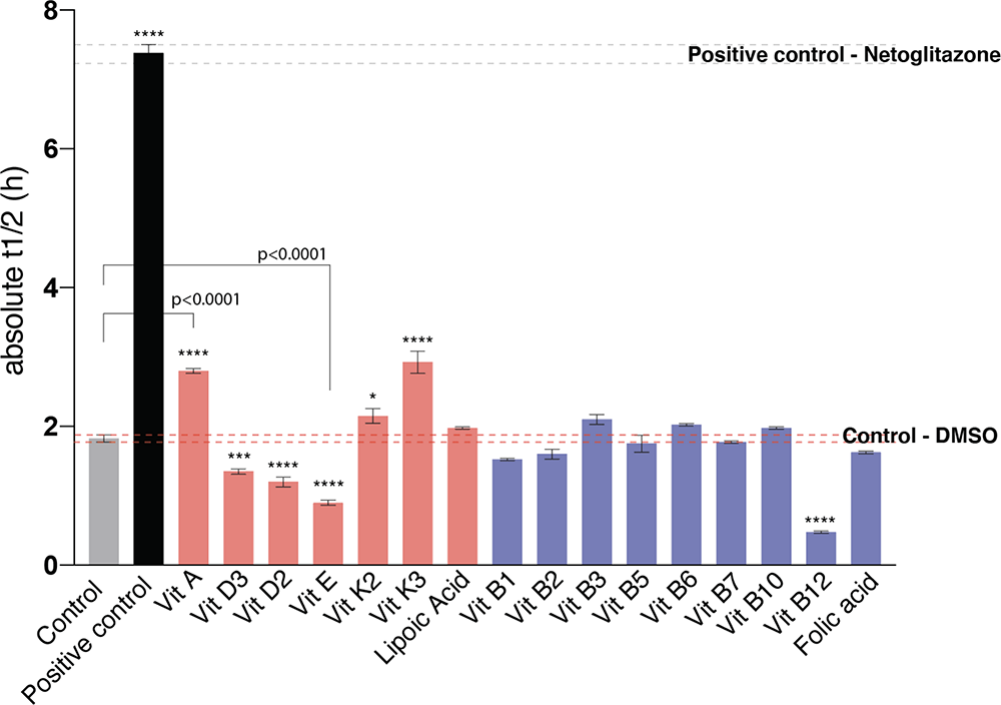
In vitro screening to identify vitamin metabolites that modulate Aβ42 aggregation. We tested human endogenous fat-soluble (red) and water-soluble (blue) vitamin metabolites in a ThT-based screen to identify vitamin metabolites that either accelerate or delay the aggregation of Aβ42. By estimating the change in half-time of aggregation, compared to the negative control DMSO and positive control netoglitazone,^21^ we identified seven vitamin metabolites as modulators of Aβ42 aggregation. The half-time is defined as the time at which the ThT signal reaches half of its final plateau value and is dependent on the initial monomer concentration. In the screen, the concentration of Aβ42 was 2 μM and that of vitamin metabolites was 20 μM. Plots are representative of three technical replicates, and we repeated the screening two times. Error bars indicate the standard error of the mean (SEM). Statistics were performed using ordinary one-way ANOVA Dunnett’s multiple-comparison test (****p-value <0.0001; ***p-value = 0.0005; *p-value = 0.03).

We carried out an in vitro aggregation screen based on thioflavin T (ThT), an amyloid-sensitive fluorescent dye (“Materials and Methods”). Our goal was to identify vitamin metabolites that modulate—either by accelerating or by inhibiting—the aggregation of Aβ42 (Figure 1). We considered 18 fat-soluble and water-soluble vitamin metabolites due to their reported dysregulation in AD^16^ (Table 1).

We found several vitamin metabolites that altered the kinetics of Aβ42 aggregation in our screen at a 20 μM metabolite concentration (10 molar equivalents, ME) compared to the negative control DMSO and positive control netoglitazone.^21^ At this concentration, these vitamin metabolites altered the half-time (*t*_1/2_) of Aβ42 aggregation, which is the time at which the ThT signal reaches half of its final plateau value (Figure 1). We found three vitamin metabolites (K2, K3, and A, see Table 1) that significantly delayed Aβ42 aggregation and four vitamin metabolites (B12, D2, D3, and E, see Table 1) that accelerated it in a statistically significant manner (Figure 1). Although we found other vitamin metabolites in our screen with smaller effects on *t*_1/2_ (Figure 1), we focused on the seven with the greatest observed effects.

**Figure 2:**
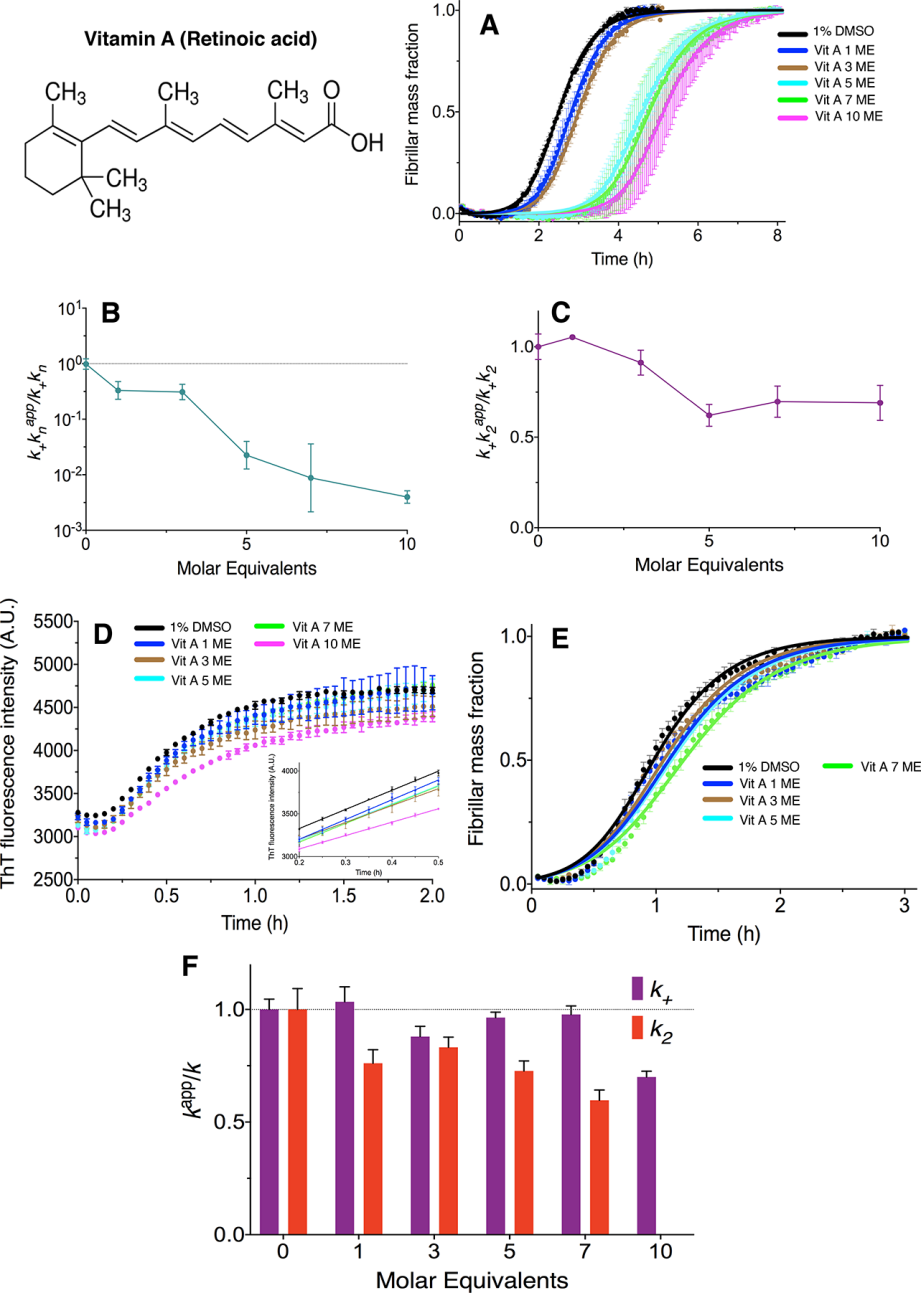
Retinoic acid inhibits the aggregation of Aβ42 by affecting both primary and secondary nucleation. (A) In an unseeded kinetic assay, we observed a dose-dependent inhibition of Aβ42 (in 1% DMSO) in the presence of retinoic acid at 1, 3, 5, 7, and 10 molar equivalents (ME). (B, C) An analysis of the changes in the rate constants from the kinetic analyses in (A) with increasing concentrations of retinoic acid shows a decrease in both primary (*k*_+_*k_n_*) and secondary (*k*_+_*k*_2_) processes. (D) In high-seeded (30% fibrils) kinetic assays, elongation is affected only at the highest concentration of retinoic acid (10 ME) (inset in D), indicating a small effect on *k*_+_. (E, F) We obtained the decrease in *k*_2_ by performing the experiments in low-seeded (2%) conditions at 1 to 7 ME concentrations, where elongation is not affected. In (F), we did not fit *k*_2_ at 10 ME, as retinoic acid affects elongation at this high concentration, preventing the decoupling through the low-seeded assay of the effects of elongation and secondary nucleation. All fits were done using AmyloFit.^37^ Plots are representative of three technical replicates, and we performed this assay two times.

**Figure 3:**
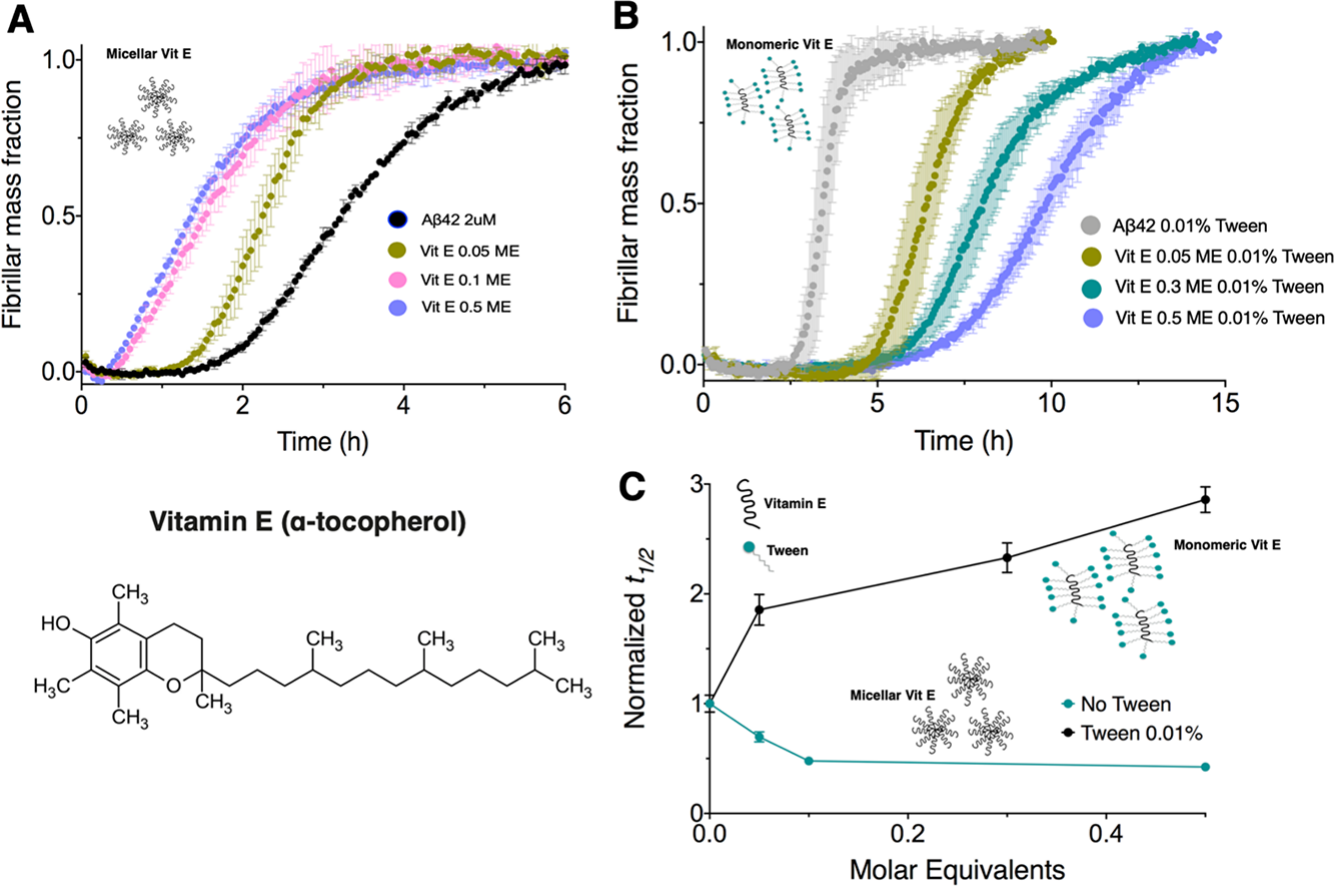
α-Tocopherol in a micellar form accelerates Aβ42 aggregation and inhibits it in a monomeric state. (A, C) We observed that α-tocopherol speeds up Aβ42 aggregation in a concentration-dependent manner when in a micellar form. (B, C) α-Tocopherol in a monomeric form (in the presence of Tween 0.01%) inhibits the aggregation of Aβ42 in a concentration-dependent manner. Plots are representative of three technical replicates, and we performed this assay two times.

We next characterized each of these vitamin metabolites by individually carrying out the kinetic analysis at lower concentrations, which may be physiologically more relevant as intra- and extracellular concentrations of vitamins can be in the nanomolar to micromolar range. We found that at low concentrations (1.2−2.8 μM), the metabolites of vitamin K2, K3, D2, D3, and B12 did not show significant effects (Figure S1), while the vitamin A (Figure 2) and E (Figure 3) metabolites induced reproducible and robust effects on Aβ42 aggregation.

### A Vitamin A Metabolite Inhibits Aβ42 Aggregation by Affecting Both Primary and Secondary Nucleation

The main microscopic processes that govern the aggregation of Aβ42 are as follows:^4,22−24^ (1) primary nucleation, which depends on the concentration of free monomers and proceeds with a rate constant *k_n_*; (2) secondary nucleation, which depends both on the concentration of free monomers and on the concentration of aggregate mass and proceeds with a rate constant of *k*_2_; and (3) elongation, where free monomers add to the growth-competent ends of existing fibrils and proceed with a rate constant *k*_+_. We observed the dose-dependent inhibition of Aβ42 in the presence of retinoic acid, a vitamin A metabolite (Table 1), in an unseeded kinetic assay (Figure 2A). Fitting the data using AmyloFit^14^ shows that there is a decrease in both *k*_+_*k_n_* and *k*_+_*k*_2_ (Figure 2B,C). We next decoupled the effects of *k*_+_ and *k_n_* in the combined rate constants. Using high-seeded kinetics (30% seed fibrils), we used the growth rate at early timescales (Figure 2D inset) to reveal effects on *k*_+_,^22^ finding that it was only overtly affected at 10 ME (20 μM) of retinoic acid (Figure 2D). Thus, for lower ME of retinoic acid (i.e., 1 to 7 ME; 2 to 14 μM), the dose-dependent inhibition of primary nucleation observed in the unseeded kinetics is likely due to a decrease in *k_n_* (Figure 2B,E). Next, we confirmed the decrease in *k*_2_ by performing the experiments in low-seeded conditions (2% seed fibrils), at ME where elongation is not significantly affected (1 to 7 ME, Figure 2E). We observed that the kinetics of Aβ42 aggregation are also inhibited under these conditions (Figure 2E,F). Note that we did not fit the 10 ME results in Figure 2F, as retinoic acid affects elongation at this high concentration, preventing the decoupling through the low-seeded assay of the effects of elongation and secondary nucleation. Taken together, our data suggest that retinoic acid inhibits the aggregation of Aβ42 predominantly by a combined effect on the primary and secondary nucleation.

### A Vitamin E Metabolite Has Opposite Effects on Aβ42 Aggregation in Its Monomeric and Micellar Forms

**Figure 4:**
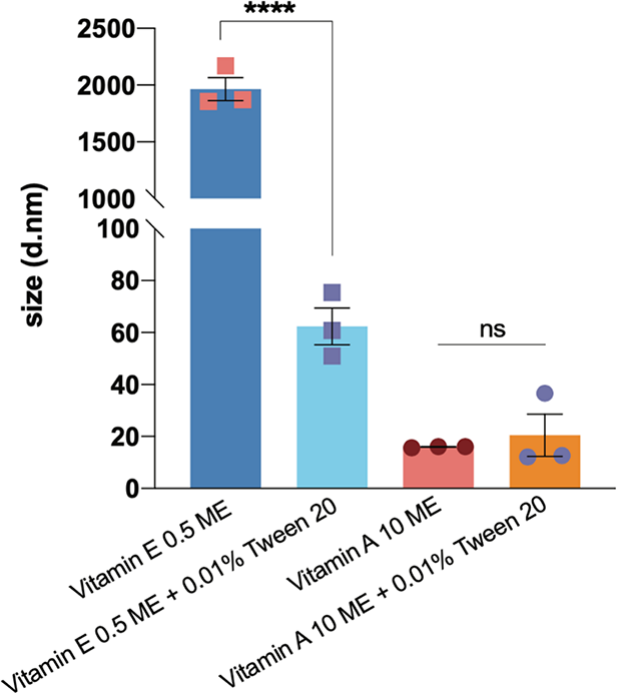
Dynamic light scattering (DLS) shows that α-tocopherol at 1 μM (corresponding to 0.5 ME in the kinetic assay) forms large aggregates which can be broken down by the addition of 0.01% Tween 20. By contrast, the size of retinoic acid in solution is not affected by the addition of Tween 20. Statistical comparisons are shown using an unpaired *t*-test, *p*-value ****<0.0001. Data show three independent replicates.

We next quantified the effects of α-tocopherol, a vitamin E metabolite (Table 1), on Aβ42 aggregation. We observed that when α-tocopherol was added in DMSO alone, it sped up the aggregation of Aβ42 (Figure 3A,C). Using dynamic light scattering (DLS), we observed that in the absence of Aβ42, α-tocopherol forms large aggregates (Figure 4) in such conditions, a process that can provide a surface promoting the aggregation of Aβ42. However, when α-tocopherol is added in the presence of Tween 20 (0.01%), which dissolves the larger assemblies, we observed that it inhibits the aggregation of Aβ42 (Figure 3B,C). For comparison, when we added Tween 20 (0.01%) to the stock solution of retinoic acid to break down potential higher-order metabolite assemblies, we did not find any differences in the inhibition of Aβ42 (Figure S2). In this context, we observed that 0.01% Tween 20 does not have effects on Aβ42 aggregation (Figures S3, S4) DLS data confirmed the breakdown of the large aggregates formed by α-tocopherol upon the addition of 0.01% Tween 20 (Figure 4). Indeed, a combination of accelerating and inhibitory properties of α-tocopherol depending on its micellar or monomeric states, respectively, is observed. In this context, we did not attempt to understand the effects of α-tocopherol on the individual microscopic steps of Aβ42 aggregation.

### Combining Retinoic Acid and α-Tocopherol Has a Near-Zero Net Effect on Aβ42 Aggregation

**Figure 5:**
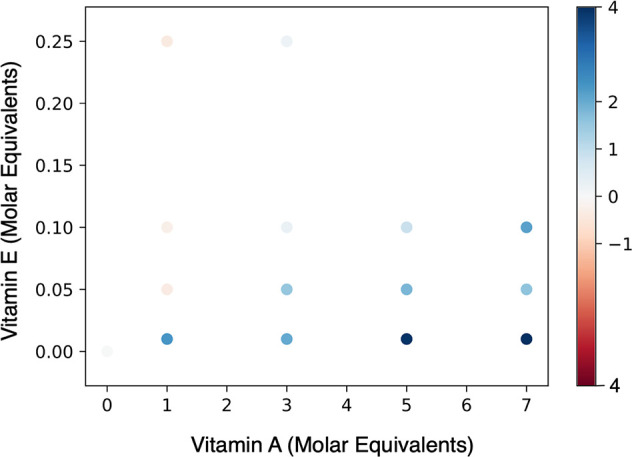
Heat map of the half-time (*t*_1/2_) of Aβ42 aggregation of different mixtures of retinoic acid (vitamin A) and α-tocopherol (vitamin E). The *x*-axis shows the molar equivalents of retinoic acid added to the molar equivalents of α-tocopherol on the *y*-axis. The map shows the range of concentrations at which the individual effect of each vitamin metabolite is canceled out by the other, thus having a near-zero net effect (see the color scale) on Aβ42 aggregation.

We next carried out the Aβ42 ThT-based aggregation assays in the presence of a mixture of retinoic acid and α-tocopherol. We thus observed that the two vitamin metabolites canceled out their individual effects (Figure 5). Although α-tocopherol in micellar form at high concentrations accelerates Aβ42 aggregation (Figures 3 and 4), we found that the strong inhibitory role of retinoic acid subdued this effect even at some of the high α-tocopherol concentrations. After testing for a combination of concentrations in a mixture, we found a concentration range (0.15–0.25 ME α-tocopherol and 0–7 ME retinoic acid) within which the net effect of retinoic acid and α-tocopherol was close to zero on the aggregation profile of Aβ42 (Figure 5).

**Figure 6:**
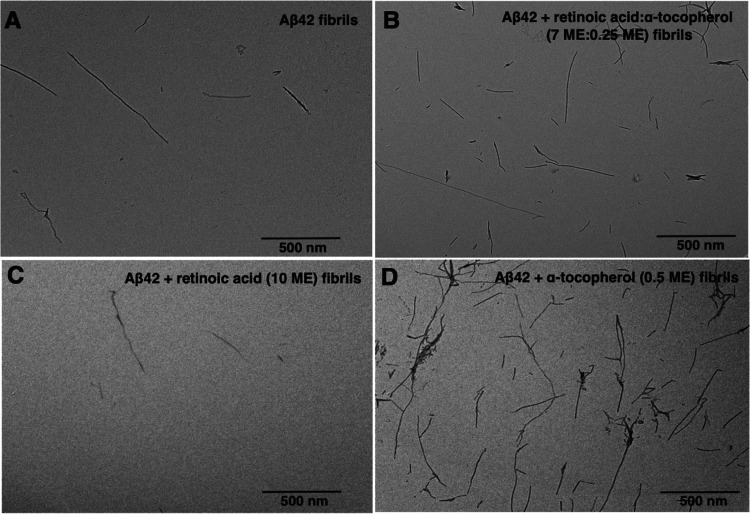
Transmission electron microscopy (TEM) images of Aβ42 fibrils formed in the presence of vitamin metabolites studied in this work. The images reveal the appearance of a heterogenous population of Aβ42 fibrils after 4 h incubation at 37 °C in 20 mM sodium phosphate buffer in the absence or presence of the two vitamin metabolites and their mixture. (A) Aβ42 alone, (B) Aβ42 + α-tocopherol (vitamin E) and retinoic acid (vitamin A) mixture (retinoic acid:α-tocopherol::7 ME:0.5 ME), (C) Aβ42 + retinoic acid (10 ME), and (D) Aβ42 + α-tocopherol (0.5 ME). Images are representative of three observations; three images were taken at different points on the grid. Scale bars represent 500 nm.

To confirm these results, we carried out transmission electron microscopy (TEM) experiments on samples deposited on carbon grids in the plateau region (*t* = 4 h) of the aggregation of monomeric Aβ42 in the absence or presence of retinoic acid and α-tocopherol (Figure 6). We observed a reduction of Aβ42 fibrils in the presence of retinoic acid (10 ME) and an increase of Aβ42 fibrils in the presence of α-tocopherol (0.5 ME) (Figure 6). In the presence of both retinoic acid and α-tocopherol (retinoic acid:α-tocopherol, 7 ME:0.25 ME), we observed Aβ42 fibrils that qualitatively resembled those formed in the absence of vitamin metabolites.

This result suggests that, taken together, the effects of these two vitamin metabolites on Aβ42 aggregation are neutralized when present in combination at a specific range of concentrations. Although here we show only two vitamin metabolites exhibiting such an effect, we expect other aggregation promoters and inhibitors to be found that would act similarly in solution. In a proof of principle, we thus propose that the complex metabolite composition of the cellular environment could have a protective role against protein aggregation through the simultaneous presence of aggregation promoters and inhibitors. The breakdown of this metastable equilibrium in metabolite composition and protein homeostasis can thus lead to protein aggregation.

### Retinoic Acid and α-Tocopherol Increase the Fitness of a *Caenorhabditis elegans* Model of Aβ42 Toxicity

**Figure 7:**
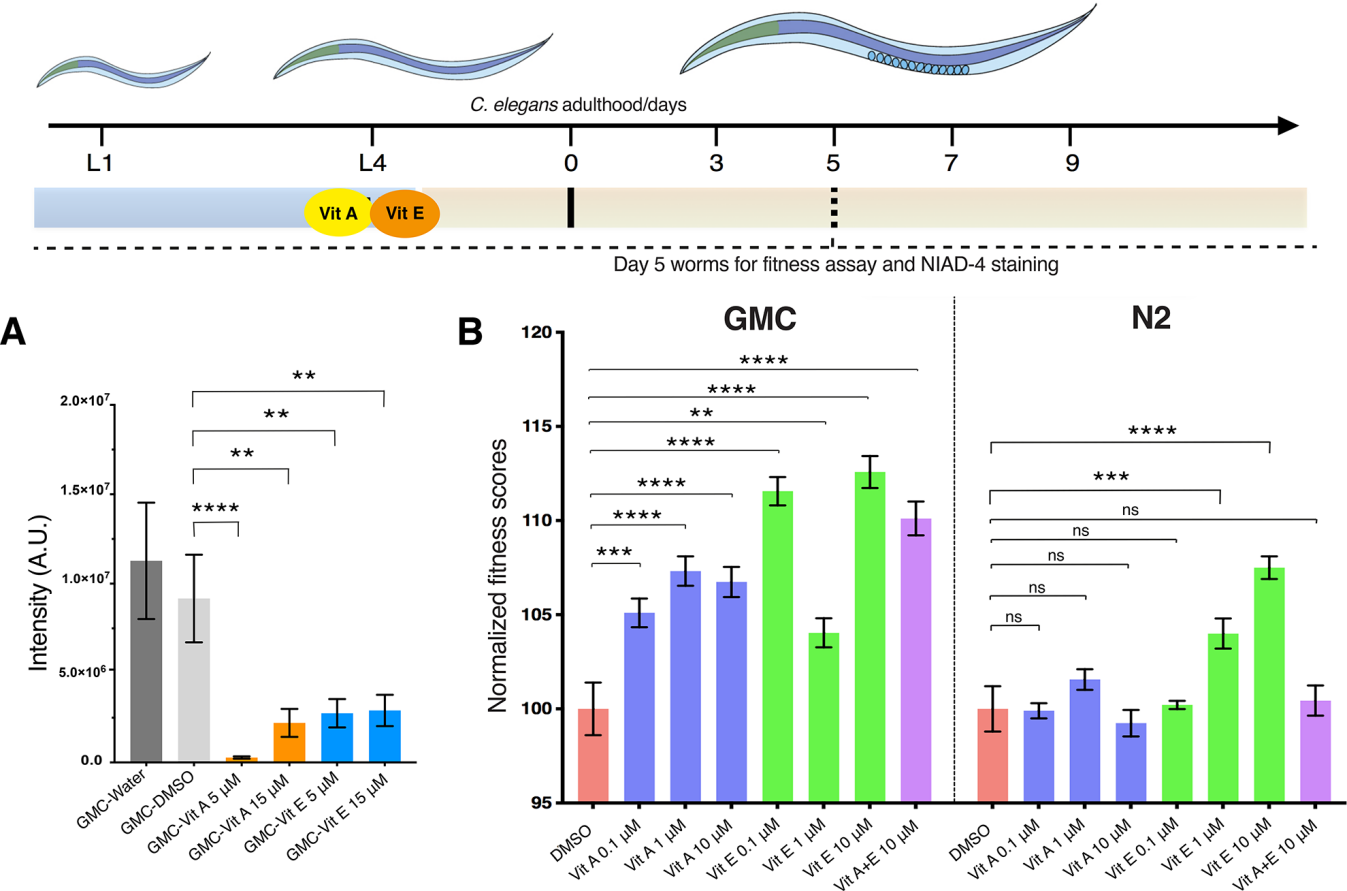
Retinoic acid and α-tocopherol decrease Aβ42 aggregate levels and improve the fitness of a *C. elegans* model of Aβ42 toxicity. We fed with retinoic acid and α-tocopherol L4 worms (GMC strain) that express Aβ42 in the muscle cells. We observed the effects of the vitamin metabolites on the motility of the animals and quantified the aggregate levels by NIAD-4 staining on day 5 of adulthood. (A) The intensity of the NIAD-4 staining shows a reduction of aggregates at both 5 and 15 μM of retinoic acid and α-tocopherol, as compared to the control worms treated with water or 1% DMSO. Wild-type N2 worms do not exhibit aggregates (not shown in the panel). (B) We treated both wild-type N2 and GMC worms with retinoic acid and α-tocopherol and tracked body bends per minute (BPMs) at day 5. In all cases, we found that the treatment with either of the two vitamin metabolites increased the fitness of the GMC worms relative to the DMSO control, while the effect was much more limited in the control N2 worms. Data were independently normalized to DMSO controls for GMC and N2 worms, respectively. All error bars represent the standard error of the mean (SEM). Statistics were preformed using one-way ANOVA, Dunnett’s multiple-comparison test, against the DMSO group, ****p < 0.0001; ***p < 0.001; **p < 0.01; *p < 0.05; ns, not significant.

To study the effects of retinoic acid and α-tocopherol on the aggregation of Aβ42 in an in vivo system, we used a *C. elegans* model of Aβ42 toxicity. In the GMC101 strain, Aβ42 is expressed in the body wall muscle cells, where it aggregates and results in a severe and fully penetrant, age-progressive paralysis.^25^ This gradual aggregation of Aβ42 in the body wall muscle cells causes worm paralysis, which can be quantified by a worm-tracking platform.^26,27^ We measured worm motility (body bends per minute, BPMs) at day 5 of adulthood when the aggregation phenotype is highly visible.^23,26^ Simultaneously, using NIAD-4, which is a dye that specifically binds mature amyloid species, we measured Aβ42 aggregates in the worm heads^10,23^ (“Materials and Methods”) and found a decrease in the NIAD-4-stained aggregates for both retinoic acid and α-tocopherol (Figure 7A). This reduction of Aβ42 aggregates upon treatment with retinoic acid and α-tocopherol corresponded to an increase in the total fitness (fitness is considered as a total of bends per minute, speed (mm/s) and live ratio) of the worms compared to the untreated worms (Figure 7B).

### Non-Specific Effects Contribute to the Reduction by Retinoic Acid of Aβ42 Oligomer Cytotoxicity

We further investigated using tissue culture experiments how retinoic acid and α-tocopherol might impact the cytotoxicity of Aβ42 oligomers, given the central role that these species play in AD.^28^ Aβ42 oligomers were stabilized as previously described,^29^ in the presence of 0.25 or 0.50 ME of α-tocopherol or 5 and 10 ME of retinoic acid. These vitamin metabolites were added from the monomeric state to assess their impact on the Aβ42 oligomer stabilization process and resulting cytotoxicity. Aβ42 oligomers reduced the health of SH-SY5Y cells to 66 ± 2%, as measured by an MTT reduction assay (see “Methods”), indicating a cytotoxic effect relative to untreated cells (Figure S5). The toxicity of Aβ42 oligomers incubated under the described conditions formed in the presence of α-tocopherol was not significantly different from the Aβ42 oligomers (one-way ANOVA relative to cells treated with Aβ42 oligomers). We observed, however, a significant increase in cell health to 97 ± 6% for Aβ42 oligomers formed in the presence of a 5 ME of retinoic acid (*P* < 0.01) and 116 ± 24% for oligomers formed in the presence of 10 ME (*P* < 0.001). As control, cells were also exposed to 1.5 μM α-tocopherol and 30 μM retinoic acid to match the samples containing Aβ42 oligomers. Of the two, only retinoic acid significantly changed the health of the cells, as it increased viability to 118 ± 3% relative to untreated cells (*P* < 0.05 by unpaired, two-tailed Student’s *t*-test; Figure S5). Collectively, these data suggest that the presence of retinoic acid can impact the cytotoxicity of Aβ42 oligomers also through processes not directly related to the Aβ42 oligomers themselves. Together with the *C. elegans* experiments, these data suggest that vitamins can modulate complex endogenous pathways to cause resistance to Aβ42 oligomers beyond a direct effect on Aβ42 aggregation.

## Discussion and Conclusions

Linus Pauling in 1977 proposed that therapies aimed at improving the molecular composition of the brain may restore abnormal functions caused by disease,^30^ a concept that was later described as vitamin homeostasis in the brain.^31^ Pauling’s suggestion gave rise to the popular megavitamin therapy for disease, which has been the backbone of the global supplements industry for decades despite a lack of clear physiological evidence on health benefits. A major reason for this lack of evidence could be attributed to the absence of fully quantitative methods to mechanistically probe this hypothesis and validate its claims. In this context, we sought to investigate the potential roles of vitamins in protein aggregation.

In this work, we have shown that several vitamin metabolites can modulate Aβ42 aggregation. Except for a vitamin B12 metabolite (cobalamin, Table 1), all the vitamin metabolites that we identified as positive hits in our screen are fat-soluble. The lipid-like nature of these vitamin metabolites could be responsible for their aggregation-modifying behavior, as shown in previous studies on other types of compounds.^15,24,32,33^ Further, our results indicate that the lipid-like nature of these vitamin metabolites can lead to the formation of assemblies that in turn may accelerate the aggregation process by a variety of possible mechanisms, including by enhancing surface-catalyzed nucleation or by increasing the local concentration of Aβ42 (Figure 3).

We have found that retinoic acid inhibits the aggregation of Aβ42 in a dose-dependent manner and that α-tocopherol accelerates Aβ42 aggregation in its self-assembled form but inhibits it in its soluble monomeric form. Further, we have described how these two vitamin metabolites can have opposite effects on Aβ42 aggregation in a mixture, as we observed a zero-sum effect^15^ on Aβ42 aggregation at a range of concentrations. We suggest that these vitamin metabolites may be added as components of the protein quality control system due to their effects on protein aggregation. It is also important to point out, however, that in addition to the direct effect on Aβ42 aggregation shown here, vitamins are most likely to have a wide variety of complex additional roles in the protein homeostasis system in AD, as our results in cells and worms indicate.

In conclusion, we have shown that retinoic acid and α-tocopherol have opposite effects on Aβ42 aggregation and that these effects can cancel out in a mixture. Considered together with related conclusions on the effect of lipid membranes of mixed composition on Aβ42 aggregation,^11^ these results suggest that a complex cellular environment may have a protective role against protein aggregation through the simultaneous presence of aggregation promoters and inhibitors. When the concentrations of these regulatory cellular components become dysregulated, however, the resilience of the system may become impaired, resulting in aberrant protein aggregation. Our results thus provide an example of a systematic approach to investigate the effects of a dysregulated molecular composition on aggregation-prone proteins.

## Materials and Methods

### Chemicals

All vitamin metabolites (Table 1) were procured from Sigma and were of analytical grade and dissolved in milli-Q grade water (for water-soluble) or DMSO (for fat/lipid-soluble). Stocks were prepared in 100% DMSO and tested in the various assays at a final working concentration of 1%. The vitamin metabolite stocks were sonicated in a water bath for 2 min for effective dissolution of the solutes. All solutions were freshly prepared for each assay and filtered using Anotop 10 0.02 μm filters prior to use in the assays.

### Expression, Purification, and Preparation of Aβ42 Peptide Samples for Kinetic Experiments

Aβ42 (MDAEFRHDSGYEVHHQKLVFFAEDVGSNKGAIIGLMVGGVVIA) was expressed recombinantly in the *Escherichia coli* BL21 Gold (DE3) strain (Stratagene, California, USA) and purified as described previously.^23^ We note that the N-terminal methionine residue used in the procedure could be oxidized by those vitamin metabolites studied here that have antioxidant effects. However, we did not observe systematic effects due to this possible effect. In the purification procedure, the *E. coli* cells were sonicated and the inclusion bodies were subsequently dissolved in 8 M urea. A diethyl-aminoethyl cellulose resin was then used to perform ion exchange chromatography, and the protein collected was lyophilized. These fractions were then further purified using a Superdex 75 26/60 column (GE Healthcare, Illinois, USA), and the fractions containing the recombinant protein were combined, frozen, and lyophilized again. Initial peptide solutions were prepared by dissolving lyophilized Aβ42 in 6 M GuHCl and purified in 20 mM sodium phosphate buffer, 200 μM EDTA, pH 8.0, using a Superdex 75 10/300 column (GE Healthcare) at a flow rate of 0.5 mL/min. ThT was added from a 2 mM stock to give a final concentration of 20 μM. All samples were prepared in low-binding Eppendorf tubes, and each sample was then pipetted into multiple wells in a 96-well half-area, low-binding, clear-bottom PEG-coated plate (Corning 3881), 80 μL per well, in the absence or presence of various concentrations of vitamin metabolites, to give a final concentration of 1% DMSO (v/v) using an Eppendorf laboratory pipetting robot.

The aggregation of Aβ42, at a 2 μM concentration,^23,24^ was initiated by placing the 96-well plate in a plate reader (FLUOstar Omega or FLUOstar Optima from BMG Labtech, Aylesbury, UK) at 37 °C under quiescent conditions. The ThT fluorescence was monitored in triplicate per sample as measured using bottom-optics with 440 nm excitation and 480 nm emission filters.

### *C. elegans* Experiments

#### Media

Standard conditions were used for the propagation of *C. elegans*.^34^ Animals were synchronized by hypochlorite bleaching (5% hypochlorite solution), hatched overnight in M9 (3 g/L KH_2_PO_4_, 6 g/L Na_2_HPO_4_, 5 g/L NaCl, 1 μM MgSO_4_) buffer, and subsequently cultured at 20 °C on nematode growth medium (NGM) (CaCl_2_ 1 mM, MgSO_4_ 1 mM, cholesterol 5 μg/mL, 250 μM KH_2_PO_4_ pH 6, Agar 17 g/L, NaCl 3 g/L, casein 7.5 g/L) plates that were seeded with the overnight grown *E. coli* strain OP50. Saturated cultures of OP50 were grown by inoculating 50 mL of LB medium (tryptone 10 g/L, NaCl 10 g/L, yeast extract 5 g/L) with OP50 and incubating the culture for 16 h at 37 °C. NGM plates were seeded with bacteria by adding 350 μL of saturated OP50 to each plate and leaving the plates at 20 °C for 2–3 days. On day 3 after synchronization, the animals were placed on NGM plates containing 5-fluoro-2′deoxy-uridine (FUdR) (75 μM) to inhibit the growth of offspring and the temperature was raised to 24 °C.

#### Strains

All strains were acquired from the Caenorhabditis Genetics Center in Minnesota, which is supported by NIH P40 OD010440. Two strains were utilized for these experiments. The temperature-sensitive human Aβ-expressing strain dvIs100 [unc-54p::A-beta-1-42::unc-54 3′-UTR + mtl-2p::GFP] (GMC101) was used, in which mtl-2p::GFP causes intestinal GFP expression and unc-54p::A-beta-1-42 expresses the human full-length Aβ42 peptide in the muscle cells of the body wall. Raising the temperature above 20 °C at the L4 or adult stage causes paralysis due to Aβ42 aggregation in the body wall muscle. The N2 strain was used for wild-type worms.

#### Vitamin-Coated Plates

Aliquots of NGM media were autoclaved and poured and seeded with 350 μL OP50 culture that was grown overnight. After incubating for up to 3 days at room temperature, aliquots of retinoic acid and α-tocopherol dissolved in 1% DMSO at different concentrations were added. NGM plates containing FUdR (75 μM) were seeded with 2.2 mL aliquots of vitamin metabolites dissolved in 1% DMSO at the appropriate concentration (Figure 7). The plates were then placed in a laminar flow hood at room temperature to dry, and the worms were transferred to plates coated with the vitamin metabolite at larval stage L4. Retinoic acid and α-tocopherol were prepared at room temperature to a stock concentration of 2 mM. α-Tocopherol was prepared at 2 mM. Fresh stocks were made for each experiment.

#### Automated Motility Assay

At different ages, animals were washed off the plates with M9 buffer and spread over an OP50 unseeded 6 cm plate, after which their movements were recorded at 20 fps using a recently developed microscopic procedure for 90 s.^26,27^ Approximately 300 animals were counted at each data point.

#### NIAD-4 Staining and Imaging

A NIAD-4 solution was prepared by dissolution in 100% DMSO at 1 mg/mL. Prior to worm incubation, a 1/1000 dilution in M9 was created. After screening using the Wide-field Nematode Tracking Platform,^26,27^ approximately 300 worms per condition were collected in M9 media and centrifuged at 20 °C at 2000 rpm for 2 min to a pellet. One milliliter of diluted NIAD-4 solution in M9 was then added to the pellet and placed under gentle shaking (80 rpm) for 6 h. Worms were then transferred to unseeded NGM plates and incubated at 20 °C for 12 h. Worms were again washed from the plates with M9 media, spun down, washed with 10 mL M9, and resuspended in 2 mL M9. After gravity sedimentation, 15 μL worm solution was spotted on 4% agarose pads. To anesthetize the animals, 4 μL of 40 mM NaN_3_ was added, followed by a glass coverslip. Worms were imaged using a Zeiss Axio Observer A1 fluorescence microscope (Carl Zeiss Microscopy GmbH, Jena, Germany) with a 20× objective and a 49004 ET-CY3/TRITC filter (Chroma Technology Corp, Vermont, USA). An exposure time of 1000 ms was employed. The nominal magnification was 40×, and images were captured using an Evolve 512 Delta EMCCD camera with high quantum efficiency (Photometrics, Tucson, Arizona, USA). Approximately 20 animals were analyzed per condition, and statistics were performed using the one-way ANOVA against the 1% DMSO group. All statistics herein were preformed using GraphPad Prism. Quantification was preformed using ImageJ (NIH, Maryland, USA) to determine the grayscale intensity mean in the head of each animal.^10^

#### Transmission Electron Microscopy (TEM)

Samples were deposited on a 400-mesh, 3 mm copper grid carbon support film (EM Resolutions Ltd., Sheffield, UK) and stained with 2% uranyl acetate (w/v). Imaging was carried out on an FEI Tecnai G2 transmission electron microscope (Cambridge Advanced Imaging Centre, University of Cambridge, UK), and the images were acquired using the SIS MegaView II Image Capture system (Olympus, Muenster, Germany). Briefly, the grid was instantaneously washed with chloroform followed by 3× Milli-Q water washes and blotted with a filter paper to remove any debris. Five microliters of sample was deposited on the grid and left for 3 min, followed by two instantaneous washes with Milli-Q water. Uranyl acetate at 2.5 μL was deposited and left for 2 min. This was wicked away using a filter paper to ensure that the patches on the grid are fully stained.

### Cell Experiments

#### SH-SY5Y Cells

Authenticated SH-SY5Y cells (ATCC, Virginia, USA) were cultured in DMEM/F-12 with l-glutamine, HEPES, and phenol red (11330032, Thermo Fisher Gibco, Massachusetts, USA) and supplemented with 10% FBS and 1.0% antibiotics (penicillin–streptomycin, Thermo Fisher Gibco, Massachusetts, USA). Cell cultures were maintained in a 5% CO_2_-humidified atmosphere at 37 °C and grown until they reached 80% confluence for a maximum of 20 passages.^29,35^ The cell line tested negative for mycoplasma contamination.

#### Oligomer Stabilization Procedure

Lyophilized Aβ42 (Sigma-Aldrich, Missouri, USA) was dissolved in 100% hexafluoro-2-isopropanol (HFIP) to 1.0 mM, and then the solvent was evaporated. Aβ-derived diffusible ligands (ADDLs) were prepared from Aβ42 solutions according to Lambert’s protocol.^36^ Briefly, Aβ42 was resuspended in high-purity DMSO to a concentration of 5 mM and diluted in phenol-red free DMEM/F-12 to a final concentration of 100 μM. After 24 h of incubation at 4 °C, the solution was centrifuged (10 min, 14,000 g, 4 °C) and the supernatant containing the oligomers was retained. The oligomer formation procedure was carried out in the presence of 1% DMSO or the indicated concentrations of vitamins A and E to assess the impact of the vitamins on the toxicity of Aβ42 oligomers under these conditions.

#### MTT Reduction Assay

Aβ42 oligomers (3 μM, in monomer equivalents) formed with or without vitamins were added to cell culture medium for 1 h at 37 °C under quiescent conditions and then added to the cell culture medium of SH-SY5Y cells seeded in 96-well plates for 24 h. All samples contained a final concentration of 1% DMSO, which was confirmed not to significantly change the viability of the untreated cells or the toxicity of Aβ42 oligomers. 3-(4,5-Dimethylthiazol-2-yl)-2,5-diphenyltetrazolium bromide (MTT) was purchased from Sigma-Aldrich, Missouri, USA, and the MTT reduction assay was performed as previously described.^29,35^ Briefly, cells were exposed to solutions of oligomers and vitamins for 24 h at 37 °C. This solution was removed (therein removing any vitamins present in the extracellular medium) and 0.5 mg/mL MTT added for 4 h at 37 °C, after which time the MTT solution was removed and replaced with DMSO for 1 h at room temperature with agitation (150 rpm). Absorbance was quantified at 590 nm using a plate reader (BioTek Synergy H1, Vermont, USA). Data shown are representative of *n* = 3 independent experiments. In each experiment, *n* = 6 technical replicates were investigated for each condition.

## Conflict of Interest

The authors declare no conflict of interest. The views expressed herein are those of the authors and do not reflect the position of the United States Military Academy, the Department of the Army, or the Department of Defense.

## Data Availability Statement

Raw data are shown within the manuscript and supplementary information. All data supporting the findings of this manuscript are available from the corresponding authors upon request.

## Supporting Information Available

Effects of selected vitamin metabolites under various conditions on Aβ42 aggregation; non-specific effects contributing to the reduction by retinoic acid of the toxicity of Aβ42 oligomers in SH-SY5Y cells (PDF)
