# Supplementary material for: Combinations of Vitamin A and Vitamin E Metabolites Confer Resilience against Amyloid-β Aggregation
Source: ACS Chem Neurosci. 2023 Feb 2;14(4):657–66. doi: 10.1021/acschemneuro.2c00523 (PMC9936541; doi:10.1021/acschemneuro.2c00523)
Supplement: Supplementary file 1 — cn2c00523_si_001.pdf [file cn2c00523_si_001.pdf]

Combinations of Vitamin A and Vitamin E Metabolites Confer Resilience Against Amyloid-β Aggregation

Priyanka Joshi[a,b,*], Sean Chia[a], Xiaoting Yang[a], Michele Perni[a], Justus M. Gabriel[c], Marshall Gilmer[c], Ryan Limbocker[c], Johnny Habchi[a] and Michele Vendruscolo[a,*]

[a]*Centre for Misfolding Diseases, Department of Chemistry, University of Cambridge, Cambridge CB2 1EW, UK*

[b]*The California Institute for Quantitative Biology (QB3-Berkeley); Department of Nutritional Sciences and Toxicology, University of California, Berkeley, CA 94720*

[c]*Department of Chemistry and Life Science, United States Military Academy, West Point, NY 10996, USA*

[*]**Correspondence to:** mv245@cam.ac.uk (MV), prijoshi@berkeley.edu (PJ)

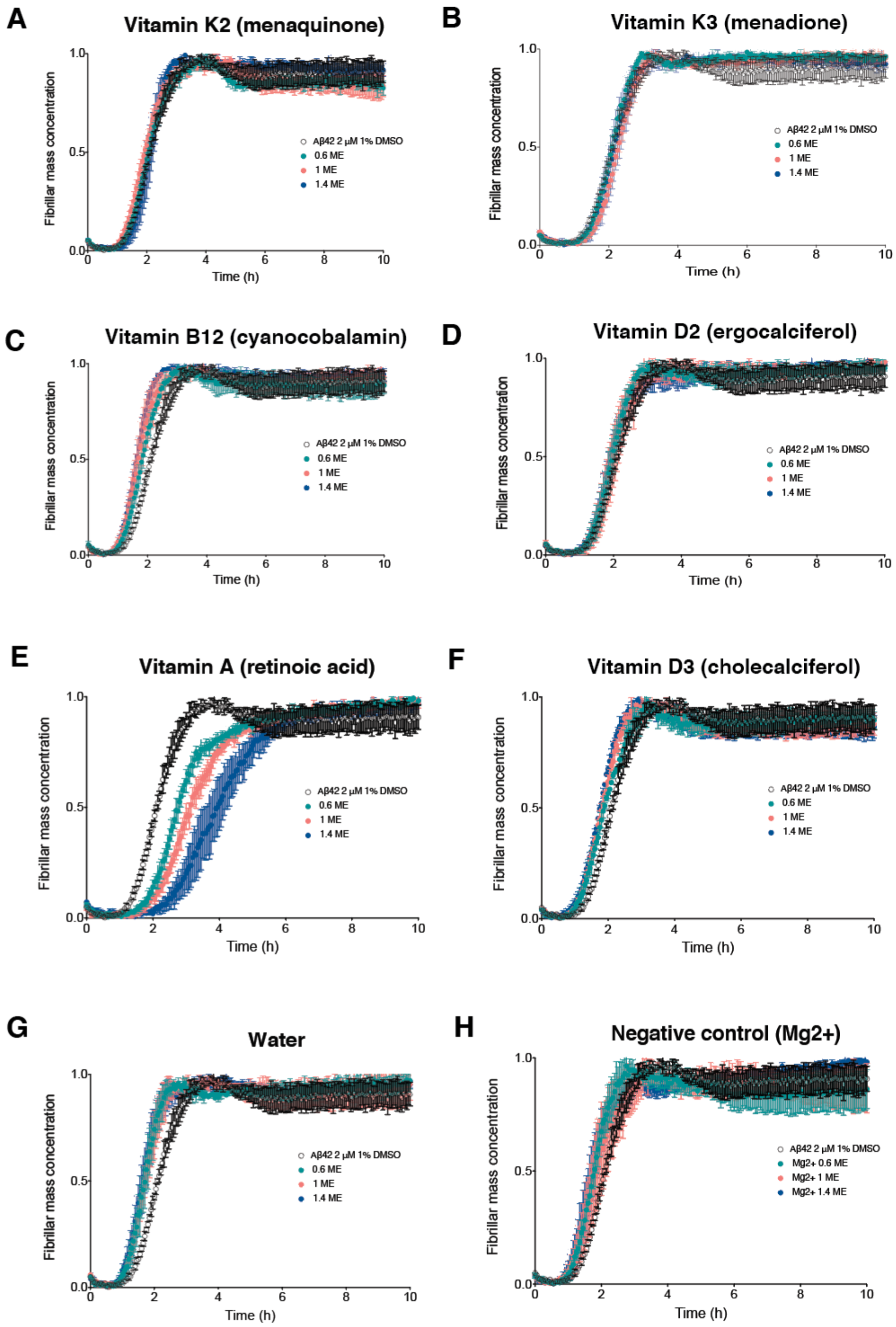

Figure S1. Effect of selected vitamin metabolites on Aβ42 aggregation. The vitamin K (A,B), B12 (C) and D (D,F) metabolites have minimal effects, and retinoic acid (E) has inhibitory effects on 2 µM Aβ42 aggregation at 0.6, 1 and 1.4 molar equivalents (ME), corresponding to 1.2, 2 and 2.8 µM respectively. Mg^{2+} is used as a negative control, as it has no significant effect on Aβ42 aggregation in this assay. Water also shows no significant effects on Aβ42 aggregation. Plots are representative of three technical replicates, and we performed this assay two times.

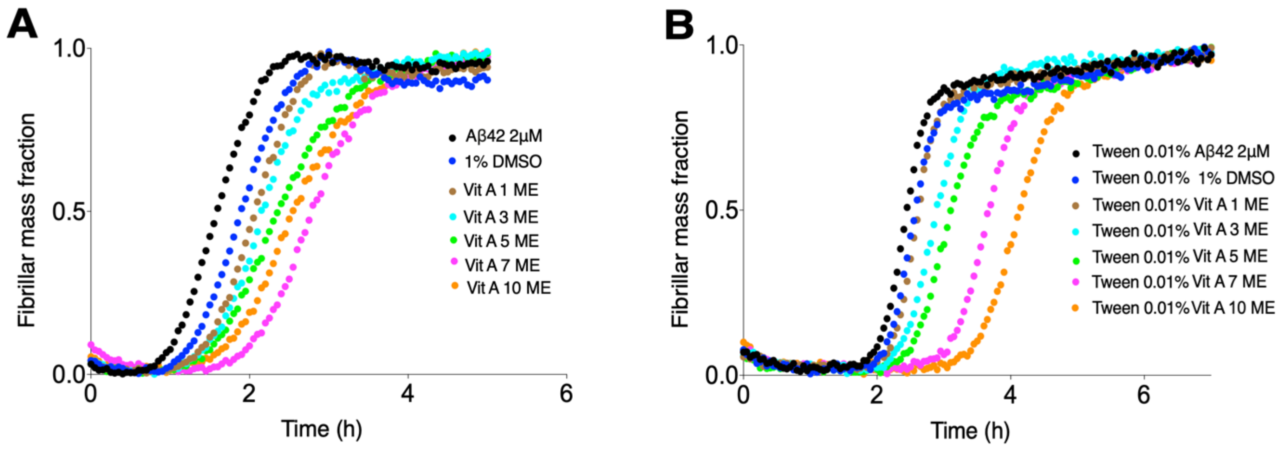

Figure S2. Effect of retinoic acid on Aβ42 aggregation in the presence of Tween 20. (A) Retinoic acid inhibits Aβ42 aggregation at 1, 3, 5, 7, 10 ME, corresponding to 2, 6, 10, 14 and 20 μM respectively. (B) On addition of Tween 20 (0.01%) to retinoic acid stock solution, we observe a similar dose-dependent inhibition of Aβ42 aggregation at 1, 3, 5, 7, 10 ME, corresponding to 2, 6, 10, 14 and 20 μM respectively. Plots are representative of three technical replicates, and we performed this assay two times.

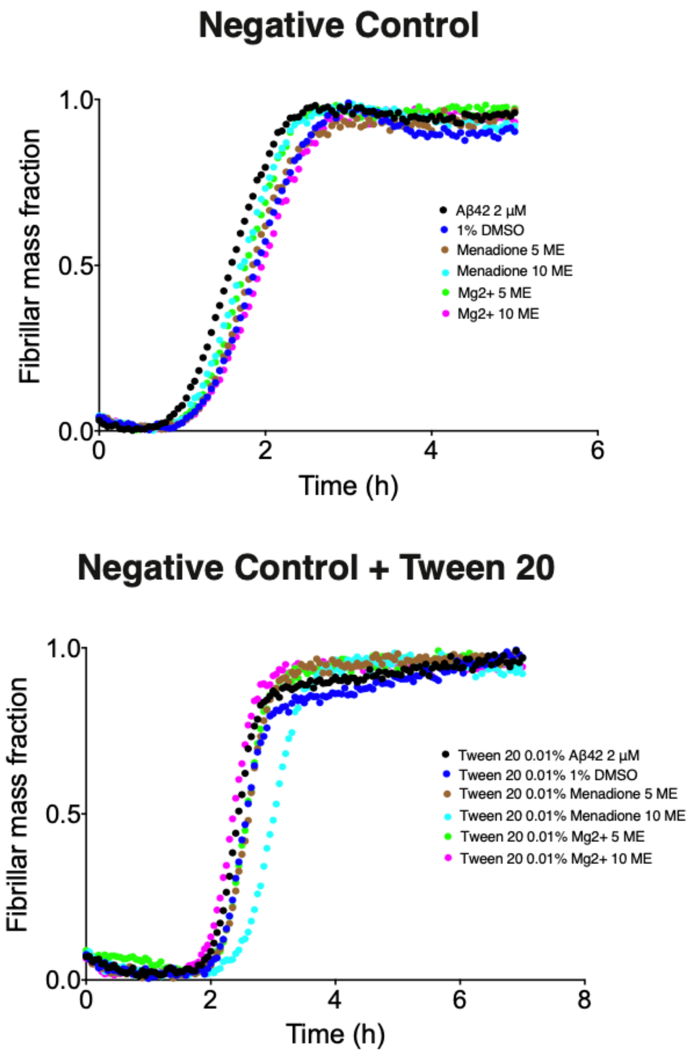

Figure S3. Effect of Tween 20, DMSO, menadione and Mg^{2+} on Aβ42 aggregation. 0.01% Tween 20 does not have any effect on Aβ42 aggregation. We show the negative controls here, 1% DMSO, menadione (Vitamin K3) and Mg^{2+}, at 5 and 10 molar equivalents (ME), corresponding to 10 and 20 μM respectively. Plots are representative of three technical replicates, and we performed this assay two times.

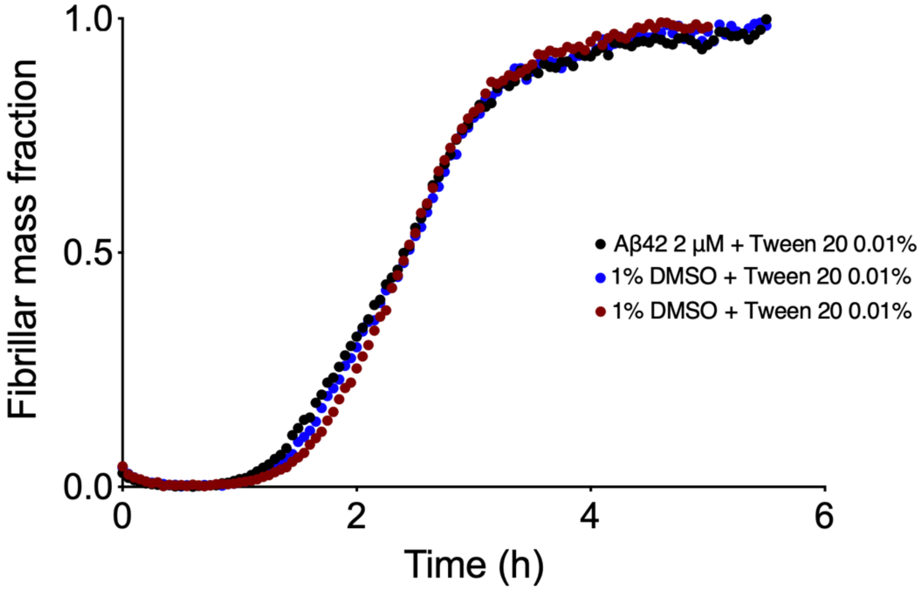

Figure S4. Effect of Tween 20 on Aβ42 aggregation. Two biological replicates show that 0.01% Tween 20 does not have any effect on Aβ42 aggregation.

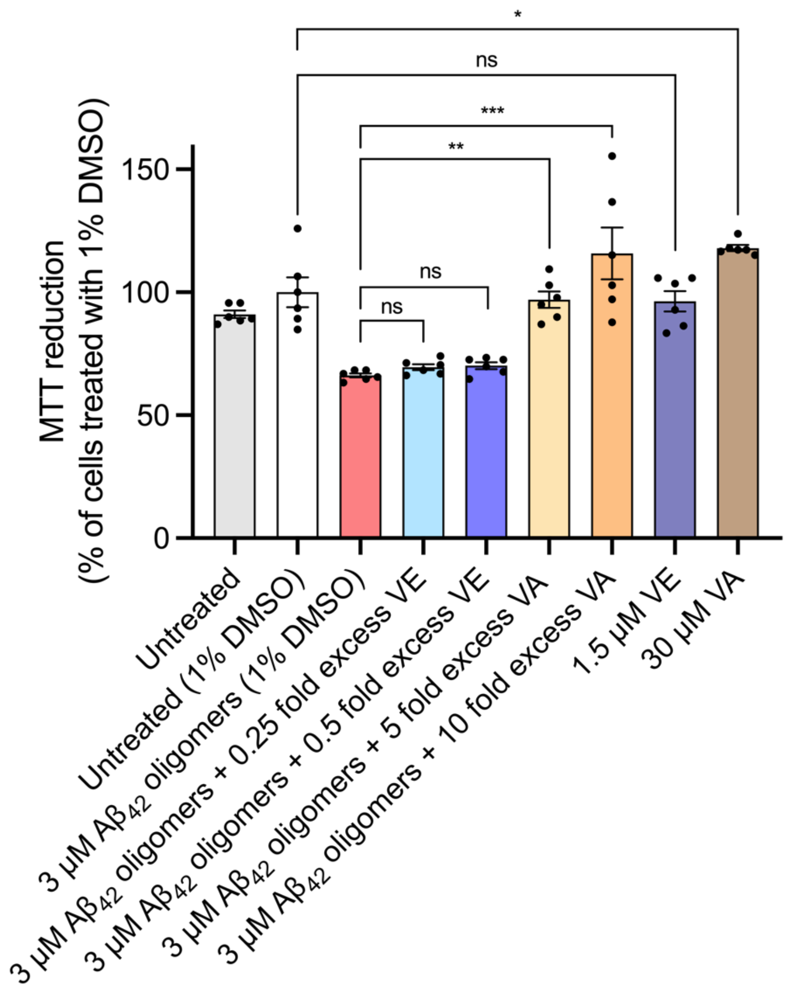

Figure S5. Non-specific effects contribute to the reduction by retinoic acid of the toxicity of Aβ42 oligomers in SH-SY5Y cells. Aβ42 oligomers were stabilized at a concentration of 100 µM as previously described[1] in the presence of either 1% DMSO, 25 µM α-tocopherol (VE), 50 µM VE, 500 µM retinoic acid (VA), or 1,000 µM VA. Vitamin metabolites were added from the monomeric state during Aβ42 oligomer stabilization. Molar ratios were selected to match the chemical kinetic experiments. After stabilization, Aβ42 oligomers were added to cell culture medium at a final concentration of 3 µM and incubated for 1 h at 37 °C under quiescent conditions, and then exposed to SH-SY5Y cells seeded in 96-well plates for 24 h. The MTT reduction assay was used to assess cell viability in the presence of 3 µM Aβ42 oligomers (red bar) and the indicated amounts of VE (blue bars) or VA (orange bars). Cells were also treated as control in the absence of oligomers and with the highest concentration of free VE (1.5 µM, dark blue bar) or VA (30 µM, brown bar) corresponding to the samples containing Aβ42 oligomers. n=60,000 cells per condition

corresponding to the six technical replicates shown. Conditions were analyzed by one-way analysis of variance (ANOVA) followed by Dunnett's multiple comparison test relative to cells treated with Aβ42 oligomers, as indicated. Untreated cells and cells treated with 1.5 μM VE or 30 μM VA were analyzed by an unpaired, two-tailed Student's t-test, as indicated. Data are representative of n=3 biologically independent experiments.

Supplementary References

1. Limbocker, R., Chia, S., Ruggeri, F. S., Perni, M., Cascella, R., Heller, G. T., ... & Dobson, C. M. (2019). Trodusquemine enhances Aβ42 aggregation but suppresses its toxicity by displacing oligomers from cell membranes. *Nature communications*, *10*(1), 1-13.
